# Prognostic Value of Combination of Pretreatment Red Cell Distribution Width and Neutrophil-to-Lymphocyte Ratio in Patients with Gastric Cancer

**DOI:** 10.1155/2018/8042838

**Published:** 2018-02-14

**Authors:** Danyang Zhou, Ying Wu, Zhenyu Lin, Liangliang Shi, Lei Zhao, Tao Liu, Dandan Yu, Tao Zhang

**Affiliations:** ^1^Cancer Center, Union Hospital, Tongji Medical College, Huazhong University of Science and Technology, Wuhan 430022, China; ^2^Department of Oncology, Second Affiliated Hospital of Nanchang University, 1 Minde Road, Nanchang, Jiangxi 330006, China

## Abstract

**Aims:**

Gastric cancer (GC) is often diagnosed at an advanced stage; inexpensive and valid biomarkers for GC are still unavailable. We aimed to evaluate the prognosis of the combination of pretreatment red cell distribution width (RDW) and neutrophil-to-lymphocyte ratio (NLR) in patients with GC.

**Methods:**

A retrospective analysis from 103 GC patients who were diagnosed at our institution from 2012 to 2016 was performed. Both pretreatment RDW and NLR were calculated based on the recommended cutoff values of 13.4% and 2.755, respectively. Combined values of RDW and NLR (RDW + NLR) stratified patients into a score of 0 (RDW ≤ 13.4% and NLR ≤ 2.755), a score of 1 (RDW > 13.4% or NLR > 2.755), and a score of 2 (RDW > 13.4% and NLR > 2.755). Prognostic significances for overall survival (OS) and progression-free survival (PFS) were assessed.

**Results:**

Pretreatment RDW + NLR was a significantly independent prognostic factor for OS and PFS. Moreover, high RDW + NLR was strongly related to age, tumor location, TNM stage, CA125, and CA199. In a subgroup analysis for patients with advanced gastric cancer (AGC), we observed that the level of RDW + NLR was markedly associated with OS and PFS.

**Conclusion:**

Pretreatment RDW + NLR is a simple, inexpensive, and valid prognostic system to predict the survival in patients with GC, especially AGC.

## 1. Introduction

Gastric cancer (GC) is the fifth most frequent cancer and the third cause of cancer-related mortalities worldwide, with 951,600 new cases diagnosed and 723,100 deaths, accounting for 6.7% and 8.8% of all cancers [1]. Eastern Asia (particularly China, Korea, and Japan) is one of the regions with the highest incidence rate [1]. A decline trend of incidence and mortality rates has been observed in GC [2, 3] which is due to the improvements in diagnoses and treatments [4–6]. Unfortunately, the 5-year survival of patients is still dismal [7–9].

Treatment strategies include surgical resection, chemotherapy, and radiotherapy which are mainly determined by TNM stage system. Nevertheless, there are still some patients with the same TNM stage and different prognosis [10]. Although a few of molecular markers are identified to stratify survival in different cohorts of GC patients [11–14], simple, inexpensive, and valid signatures to generate prognostic model are still unavailable at clinical settings.

It is well known that GC is an inflammation-associated malignancy [15]. Chronic infection is one of the strongest identified risk factors for cancers [16]. Several serum systemic inflammation biomarkers, including NLR and RDW, have been shown to possess potential to predict survival in some cancers, such as lung cancer [17, 18], breast cancer [19], and colorectal carcinoma [20]. Recently, a study has reported the prognostic values of combining RDW with NLR (RDW + NLR) to predict survival in patients with epithelial ovarian cancer [21]. However, studies regarding the prognostic values of RDW combined with NLR in patients with GC have not been reported. In this study, we retrospectively investigated the prognostic significance of pretreatment RDW + NLR in patients with GC.

## 2. Materials and Methods

### 2.1. Patients and Follow-Up

We performed a retrospective study of patients with confirmed GC by histopathology at the Cancer Center of Union Hospital, Tongji Medical College, Huazhong University of Science and Technology from 2012 to 2016. Patients meeting any of the following criteria are excluded: (1) patients with incomplete pretreatment serum parameters, (2) any malignancies besides GC, (3) hematological diseases, (4) evidences of infection or autoimmune diseases, and (5) severe complications or deaths occurred within 15 days after diagnosis.

Finally, 103 GC patients were involved and all patients underwent pretreatment evaluations. Clinical information was collected from medical records at the Cancer Center. Clinical stage of the disease was determined following the 7th American Joint Committee on Cancer (AJCC) guidelines [22]. This retrospective study was approved by the Ethical Committees of our Cancer Center. All patients were followed regularly in our institutions with tumor markers and computed tomography (CT) every 3–6 months. If there are hints of recurrence, additional investigations, such as magnetic resonance imaging (MRI) and/or positron emission tomography-computed tomography (PET-CT) procedures, were performed. Recurrence was defined as radiological evidence of intra-abdominal or abdominal soft tissue around the surgical site, or else distant metastasis. For patients who died, survival time and progression time after diagnosis were recorded. For survivors (up to January 10, 2017), time after diagnosis and recurrence status were recorded, instead.

### 2.2. Statistical Analysis

OS was defined as time from diagnosis to death (all causes) or the time the patient was last known to be alive. PFS was defined as time from diagnosis to the first progression or the time the patient was last known to be alive. The optimal cutoff value of RDW (≤13.4% and >13.4%) was defined using the median value and referred to data from previous studies [23, 24]. Analysis of receiver operating characteristic (ROC) curves was performed to identify the cutoff value of 2.755 for NLR (area under the curve (AUC) = 0.728, sensitivity = 71.9%, and specificity = 75.0%). And the cutoff values of other parameters were decided by the median values or ROC curves. Patients were categorized into three groups through the prognostic system (RDW + NLR), namely, patients with RDW ≤ 13.4% and NLR ≤ 2.755 were defined as RDW + NLR = 0, patients with RDW > 13.4% or NLR > 2.755 were defined as RDW + NLR = 1, and patients with RDW > 13.4% and NLR > 2.755 were defined as RDW + NLR = 2. Kaplan–Meier method and log-rank test were used for survival analysis on categorical variables. For continuous variables, the data were displayed as mean ± standard deviation (SD) or mean (range). Associations of RDW + NLR with other clinical pathological parameters were determined using chi-square test or Kruskal-Wallis tests according to the categories of these variables. Univariate and multivariate survival analyses were carried out using Cox proportional hazards model, and clinical pathological parameters that had significant effects on univariate analysis were subjected to multivariate analysis. All statistical analysis was performed using IBM SPSS package (Statistical Package for the Social Sciences; Version 22.0, Armonk, NY). All *P* values were two-sided, and *P* values < 0.05 were considered significant.

## 3. Result

### 3.1. Patient Characteristics and Follow-Up

A total of 103 GC patients meeting the inclusion criteria were enrolled in the present study. The median age of these patients at diagnosis was 54 years (range 27 to 80). Among them, 40.8% (42/103) were female, and 59.2% (61/103) were male. Of these patients, including 38 patients with RDW + NLR = 0, 37 patients with RDW + NLR = 1, and 28 patients with RDW + NLR = 2. The location of tumor was divided into pyloric antrum (48.5%, 50/103) and nonpyloric antrum (51.5%, 53/103). Among all GC cases, 19.4% (20/103) were stage I-II, 24.3% (25/103) were stage III, and 56.3% (58/103) were stage IV. As for laboratory characteristics, mean RDW and NLR were 13.4% (range 11.5 to 32.7) and 2.54 (range 1.00 to 32.28), respectively (Table 1).

The median follow-up time was 8.9 months and the median OS and PFS were 8.9 months and 6.1 months, respectively. Among the three patient groups, the median OS and PFS of RDW + NLR = 0 group were 17.4 months and 11.3 months. These results were significantly higher compared with the other groups in which the median OS and PFS were 8.3 months and 6.7 months (RDW + NLR = 1) and 5.3 months and 4.7 months (RDW + NLR = 2), respectively.

### 3.2. Associations of RDW + NLR with Other Clinical Pathological Parameters

In analyzing the correlation between RDW + NLR and clinical pathological factors, significant differences were found among RDW + NLR groups, including age (*P* = 0.041), tumor location (*P* = 0.014), TNM stage (*P* = 0.007), and metastasis (*P* = 0.001). There were prognostic significances among the three groups in RDW (*P* < 0.001), WBC (*P* = 0.001), NLR (*P* < 0.001), and tumor markers, including CA125 (*P* = 0.022) and CA199 (*P* = 0.042) (Table 2).

### 3.3. RDW + NLR Has Independently Prognostic Significance

According to the cutoff values of clinical pathological variables, patients were separated into diverse groups (Table 2). To determine the optimal marker for GC patient prognosis, we investigated the prognostic value of RDW combined with NLR. Univariate analysis demonstrated that RDW + NLR was found to have high prognostic value (HR: 3.252, 95% CI: 1.289–8.203, and *P* = 0.001) for OS (Table 3). Besides, RDW (HR: 3.497, 95% CI: 1.713–7.140, and *P* = 0.001), NLR (HR: 6.482, 95% CI: 3.131–13.418, and *P* < 0.001), WBC (HR: 2.165, 95% CI: 1.084–4.324, and *P* = 0.029), monocyte count (HR: 2.125, 95% CI: 1.067–4.232, and *P* = 0.032), and CA125 (HR: 2.241, 95% CI: 1.128–4.453, and *P* = 0.021) were significantly associated with OS in univariate analysis (Table 3).

Similar results were revealed in the relationships of these factors with PFS, and RDW + NLR was markedly prognostic in PFS (HR: 1.923, 95% CI: 0.941–3.927, and *P* < 0.001) (Table 4). Multivariate analysis demonstrated that pretreatment RDW + NLR was significantly correlated with OS (HR: 3.197, 95% CI: 1.248–8.191, and *P* < 0.001) (Table 3) and PFS (HR: 2.016, 95% CI: 0.982–4.136, and *P* < 0.001) (Table 4).

**Figure 1:**
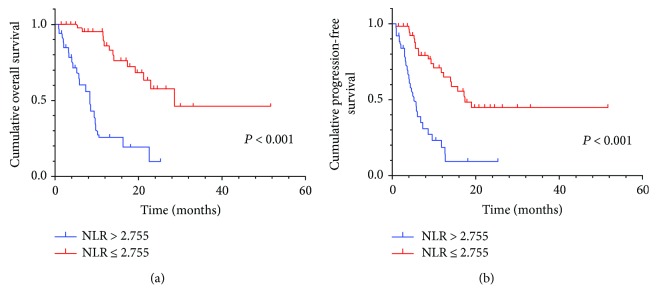
(a) Kaplan–Meier curve for OS of 103 GC patients stratified by NLR. Median OS is 5.8 (95% CI: 5.2–9.4) and 13.0 (95% CI: 12.2–17.7) for NLR > 2.755 and NLR ≤ 2.755, respectively. (b) Kaplan–Meier curve for PFS of 103 GC patients stratified by NLR. Median PFS is 4.7 (95% CI: 4.3–7.5) and 8.9 (95% CI: 9.7–14.8) for NLR > 2.755 and NLR ≤ 2.755, respectively.

**Figure 2:**
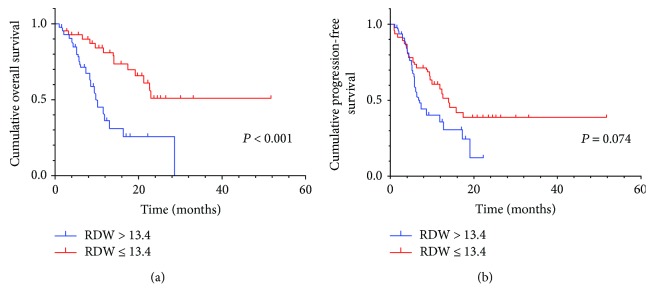
(a) Kaplan–Meier curve for OS of 103 GC patients stratified by RDW. Median OS is 5.7 (95% CI: 6.3–10.0) and 14.1 (95% CI: 13.0–19.4) for RDW > 13.4 and NLR ≤ 13.4, respectively. (b) Kaplan–Meier curve for PFS of 103 GC patients stratified by RDW. Median PFS is 5.3 (95% CI: 5.5–8.5) and 9.5 (95% CI: 9.6–15.7) for RDW > 13.4 and NLR ≤ 13.4, respectively.

**Figure 3:**
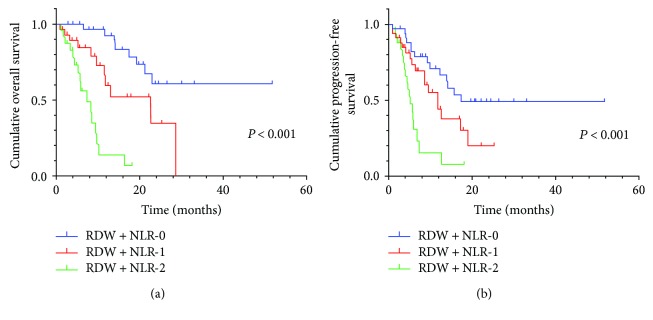
(a) Kaplan–Meier curve for OS of 103 GC patients stratified by RDW + NLR. Median OS is 17.4 (95% CI: 14.1–21.4), 9.6 (95% CI: 7.1–12.8), and 5.3 (95% CI: 4.4–7.9) for RDW + NLR = 0, RDW + NLR = 1, and RDW + NLR = 2, respectively. (b) Kaplan–Meier curve for PFS of 103 GC patients stratified by RDW + NLR. Median PFS is 11.3 (95% CI: 10.6–18.1), 6.7 (95% CI: 6.3–10.7), and 4.7 (95% CI: 3.6–6.5) for RDW + NLR = 0, RDW + NLR = 1, and RDW + NLR = 2, respectively.

By Kaplan–Meier analysis and corresponding log-rank test, we observed the high NLR had a more probability of poor OS and PFS than the low group (Figure 1). Similarly, high RDW also predicted a low survival, not progression (Figure 2). Among the three RDW + NLR groups, significant differences in OS (*P* < 0.001) and PFS (*P* < 0.001) were expressed (Figure 3), namely, patients with RDW + NLR = 2 had poorer prognoses than those with RDW + NLR = 0 or 1. Therefore, we could clearly classify the patients with GC into three independently prognostic groups.

### 3.4. Subgroup Analyses

**Figure 4:**
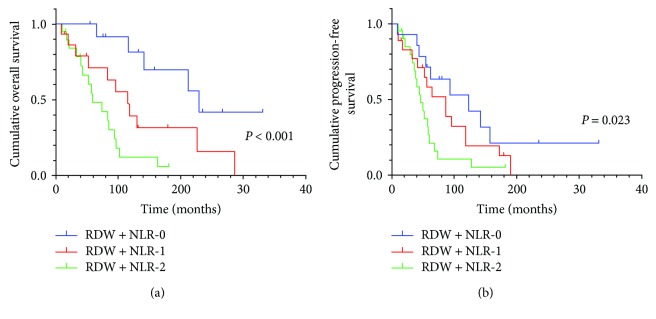
(a) Kaplan–Meier curve for OS of 58 AGC patients stratified by RDW + NLR. Median OS is 14.1 (95% CI: 10.9–21.4), 8.3 (95% CI: 5.8–14.7), and 5.4 (95% CI: 4.2–8.6) for RDW + NLR = 0, RDW + NLR = 1, and RDW + NLR = 2, respectively. (b) Kaplan–Meier curve for PFS of 58 AGC patients stratified by RDW + NLR. Median PFS is 7.8 (95% CI: 5.7–15.7), 6.0 (95% CI: 4.8–10.6), and 4.4 (95% CI: 3.3–6.9) for RDW + NLR = 0, RDW + NLR = 1, and RDW + NLR = 2, respectively.

In here, given that 56.3% patients were with AGC, the subgroup analysis on AGC were performed. Subgroup analysis showed RDW + NLR had a predictive value in OS and PFS (*P* < 0.001, *P* = 0.023, resp.), and the lower RDW + NLR score was inclined to have a better prognosis than the higher RDW + NLR score (Figure 4).

## 4. Discussion

Inflammation has been identified as one of the hallmarks of several human cancers [25]. Increasing evidence indicates that tumor-associated inflammation and tumor microenvironment play a more and more important role in the cancer development, progression, metastasis [26–29], and clinical prognosis [30–32]. As reported previously, NLR and RDW were closely related to the prognosis in several types of cancers [17, 33, 34]. In the present study, we also observed that elevated NLR, RDW, and RDW + NLR indicated poorer OS and/or PFS. Besides, in analyzing the correlation between factors of interest and other clinical pathological variables, RDW + NLR score was higher in patients with higher tumor burden and more advanced TNM stage which indicated poorer survival. Therefore, it is reasonable to combine RDW and NLR as a scoring system to enrich the stratification of prognosis in GC patients. Furthermore, we carried out the subgroup analysis on AGC patients and the results of AGC patients were similar to that of GC patients.

The elevation of NLR usually means neutrophilia and lymphocytopenia. Tumor-associated neutrophils (TANs) and other cells such as phagocytes produce a variety of cytokines and cytotoxic mediators [35–40] which play a vital role in promoting angiogenesis, stimulating DNA damage, rebuilding the extracellular matrix (ECM) to facilitate invasion, and evading host defense mechanisms. On the contrary, CD8+ cytotoxic T lymphocytes contribute to the tumor-specific adaptive immunity by attacking tumor cells [41, 42]. Moreover, several studies have been reported that neutrophilia as an inflammatory response inhibits the cytolytic activity of immune cells and causes suppression of the immune system [43, 44]. RDW elevation is strongly associated with the increase of other inflammation markers, such as interleukin-6 and tumor necrosis factor-*α* that can affect the tumor cell biological behaviors [45, 46]. This may explain why high RDW + NLR score was significantly correlated with facets of high tumor burden, including TNM stage, metastasis, pretreatment CA125 and CA199 level, and poor OS and PFS in the current study. We also found that a higher RDW + NLR score had an older population distribution, and the consistent conclusion may result from the mechanism of metabolic aging and longevity [47, 48]. Moreover, gastric parietal cells which are mainly located in the pyloric antrum play a key role in the absorption of iron, vitamin B12, and folic acid which participate in the synthesis of hemoglobin. Previous studies have shown that increased RDW was related to decreased hemoglobin [49]. This provides an implication on the relationship between RDW + NLR and the tumor location in our study. However, the details of mechanism need further study.

This study evaluated the relationship between RDW + NLR scores and prognoses in GC patients. In our study, there were three RDW + NLR scores: RDW + NLR = 0, characterized by a probability of good prognosis; RDW + NLR = 1, characterized by a probability of medium prognosis; and RDW + NLR = 2, characterized by a probability of poor prognosis. The combination of RDW and NLR evaluates their prognostic potential in GC which helps clinicians to predict the survival of GC patients.

As mentioned above, despite this study has many clinical implications, we should be clear that it is a retrospective study with its own limitations. First, our study had a retrospective design that included 103 GC patients from a single institution. Thus, our study findings need to be validated using a larger cohort of patients and multicenter trials. Second, several studies have used different RDW and NLR cutoff values, which need to be identified.

## 5. Conclusion

Pretreatment RDW + NLR was a significantly independent prognostic factor for OS and PFS. Moreover, RDW + NLR routinely measured by automated hematology analyzer is always cost-effective, reproducible, and available. Thus, RDW + NLR score is a promising prognostic marker helpful for the clinical decision-making process regarding cancer outcomes.

## Figures and Tables

**Table 1:** General characteristics of patients.

Variable	Median (range or absolute frequency)
Age (year)	54 (27–80)
Gender (female)	42 (40.8%)
Tumor location (pyloric antrum)	50 (48.5%)
HER-2 (positive)	28 (27.2%)
TNM stage I-II/III/IV	20 (19.4%)/25 (24.3%)/58 (56.3%)
Metastasis (yes)	58 (56.3%)
RDW (%)	13.4 (11.5–32.7)
WBC (g/L)	5.69 (3.03–12.81)
MO (g/L)	0.42 (0.11–1.46)
NLR	2.54 (1.00–32.28)
CA125 (U/mL)	26.6 (4.0–4853.6)
CA199 (U/mL)	11.9 (2.0–1200.0)
CEA (*μ*g/L)	2.4 (0.5–12854.0)
RDW + NLR 0/1/2	38 (36.9%)/37 (35.9%)/28 (27.2%)
Overall survival	8.9 (0.9–51.7)
Progression-free survival	6.1 (0.9–51.7)

RDW: red cell distribution width; NE: neutrophil; MO: monocyte; PDW: platelet distribution width; NLR: neutrophil-to-lymphocyte ratio; RDW + NLR: combination of red blood cell distribution width and neutrophil-to-lymphocyte ratio.

**Table 2:** Clinical pathological characteristics between different RDW + NLR groups.

Variables	RDW + NLR = 0, *n* (%)	RDW + NLR = 1, *n* (%)	RDW + NLR = 2, *n* (%)	*P*
Age				**0.041**
≤54	24 (23.3%)	20 (19.4%)	9 (8.7%)	
>54	14 (13.6%)	17 (16.5%)	19 (18.4%)	
Gender				0.565
Female	13 (12.6%)	16 (15.5%)	13 (12.6%)	
Male	25 (24.3%)	21 (20.4%)	15 (14.6%)	
Tumor location				**0.014**
Pyloric antrum	22 (21.4%)	21 (20.4%)	7 (6.8%)	
Nonpyloric antrum	16 (15.5%)	16 (15.5%)	21 (20.4%)	
HER-2				0.867
Positive	11 (10.7%)	11 (10.7%)	6 (5.8%)	
Negative	20 (19.4%)	15 (14.6%)	10 (9.7%)	
Unknown	7 (6.8%)	11 (10.7%)	12 (11.7%)	
TNM stage				**0.007**
I-II	11 (10.7%)	8 (7.8%)	1 (1.0%)	
III	13 (12.6%)	8 (7.8%)	4 (3.9%)	
IV	14 (13.6%)	21 (20.4%)	23 (22.3%)	
Metastasis				**0.001**
Yes	14 (13.6%)	21 (20.4%)	23 (22.3%)	
No	24 (23.3%)	16 (15.5%)	5 (4.9%)	
RDW (%)				**<0.001**
≤13.4%	38 (36.9%)	15 (14.6%)	0 (0.0%)	
>13.4%	0 (0.0%)	22 (21.4%)	28 (27.2%)	
WBC (g/L)				**0.001**
≤5.69	22 (21.4%)	21 (20.4%)	9 (8.7%)	
>5.69	16 (15.5%)	16 (15.5%)	19 (18.4%)	
MO (g/L)				0.830
≤0.42	21 (20.4%)	23 (22.3%)	9 (8.7%)	
>0.42	17 (16.5%)	14 (13.6%)	19 (18.4%)	
NLR				**<0.001**
≤2.755	38 (36.9%)	22 (21.4%)	0 (0.0%)	
>2.755	0 (0.0%)	15 (14.6%)	28 (27.2%)	
CA125 (U/mL)				**0.022**
≤26.6	23 (22.3%)	22 (22.3%)	11 (10.7%)	
>26.6	15 (14.6%)	15 (14.6%)	17 (16.5%)	
CA199 (U/mL)				**0.042**
≤11.9	23 (22.3%)	19 (18.4%)	14 (13.6%)	
>11.9	15 (14.6%)	18 (17.5%)	14 (13.6%)	
CEA (*μ*g/L)				0.190
≤2.4	21 (20.4%)	22 (21.4%)	11 (10.7%)	
>2.4	17 (16.5%)	15 (14.6%)	17 (16.5%)	

RDW + NLR: combination of red blood cell distribution width and neutrophil-to-lymphocyte ratio; RDW: red cell distribution width; MO: monocyte; NLR: neutrophil-to-lymphocyte ratio. *P* less than 0.05 is statistically significant.

**Table 3:** Cox proportional hazard regression analysis of patients' overall survival.

Variables	Univariable		Multivariable	
	HR (95% CI)	*P*	HR (95% CI)	*P*
Age (≤54/>54)	1.698 (0.860–3.354)	**0.127**		
Gender (F/M)	0.551 (0.282–1.075)	0.080		
Tumor location (pyloric/nonpyloric antrum)	1.130 (0.580–2.204)	0.719		
HER-2 (positive/negative)	1.818 (0.698–4.736)	0.221		
RDW (≤13.4%/>13.4%)	3.497 (1.713–7.140)	**0.001**		
WBC (≤5.69/>5.69 g/L)	2.165 (1.084–4.324)	**0.029**	1.323 (0.584–2.997)	0.502
MO (≤0.42/>0.42 g/L)	2.125 (1.067–4.232)	**0.032**	1.205 (0.521–2.790)	0.663
NLR (≤2.755/>2.755)	6.482 (3.131–13.418)	**<0.001**		
CA125 (≤26.6/>26.6 U/mL)	2.241 (1.128–4.453)	**0.021**	1.858 (0.924–3.734)	0.082
CA199 (≤11.9/>11.9 U/mL)	1.479 (0.761–2.872)	0.248		
CEA (≤2.4/>2.4 *μ*g/L)	1.513 (0.773–2.961)	0.227		
RDW + NLR	3.252 (1.289–8.203)	**<0.001**	3.197 (1.248–8.191)	**<0.001**

HR: hazard ratio; CI: confidence interval; RDW + NLR: combination of red blood cell distribution width and neutrophil-to-lymphocyte ratio. *P* less than 0.05 is statistically significant. Univariate and multivariate analysis performed using Cox proportional hazards models. Multivariate analyses using the 5 significant variables (age, WBC, MO, CA125, and RDW + NLR, except RDW and NLR) above were performed. Significant factors in univariate and multivariate analysis are indicated in bold.

**Table 4:** Cox proportional hazard regression analysis of patients' progression-free survival.

Variables	Univariable		Multivariable	
	HR (95% CI)	*P*	HR (95% CI)	*P*
Age (≤54/>54)	1.244 (0.709–2.184)	0.446		
Gender (F/M)	0.854 (0.486–1.502)	0.585		
Tumor location (pyloric/nonpyloric antrum)	1.17 (0.667–2.053)	0.584		
HER-2 (positive/negative)	1.315 (0.614–2.816)	0.481		
RDW (≤13.4%/>13.4%)	1.672 (0.943–2.964)	0.078		
WBC (≤5.69/>5.69 g/L)	2.175 (1.227–3.856)	**0.008**	1.600 (0.781–3.277)	0.199
MO (≤0.42/>0.42 g/L)	1.870 (1.059–3.302)	**0.031**	0.987 (0.475–2.048)	0.971
NLR (≤2.755/>2.755)	4.187 (2.328–7.529)	**<0.001**		
CA125 (≤26.6/>26.6 U/mL)	1.897 (1.077–3.343)	**0.027**	1.645 (0.918–2.945)	0.094
CA199 (≤11.9/>11.9 U/mL)	1.288 (0.733–2.264)	0.379		
CEA (≤2.4/>2.4 *μ*g/L)	1.609 (0.916–2.827)	0.098		
RDW + NLR	1.923 (0.941–3.927)	**<0.001**	2.016 (0.982–4.136)	**<0.001**

HR: hazard ratio; CI: confidence interval; RDW: red cell distribution width; MO: monocyte; NLR: neutrophil-to-lymphocyte ratio; RDW + NLR: combination of red blood cell distribution width and neutrophil-to-lymphocyte ratio. *P* less than 0.05 is statistically significant. Univariate and multivariate analysis performed using Cox proportional hazards models. Multivariate analyses using the 4 significant variables (WBC, MO, CA125, and RDW + NLR, except NLR) above were performed. Significant factors in univariate and multivariate analysis are indicated in bold.
